# Pupil Response in Visual Tracking Tasks: The Impacts of Task Load, Familiarity, and Gaze Position

**DOI:** 10.3390/s24082545

**Published:** 2024-04-16

**Authors:** Yun Wu, Zhongshi Zhang, Yao Zhang, Bin Zheng, Farzad Aghazadeh

**Affiliations:** 1 Department of Surgery, Faculty of Medicine and Dentistry, University of Alberta, Edmonton, AB T6G 2S2, Canada; yun.wu@ualberta.ca (Y.W.); zszhang@ualberta.ca (Z.Z.); yao.zhang@ualberta.ca (Y.Z.); bin.zheng@ualberta.ca (B.Z.); 2 Department of Mechanical Engineering, University of Alberta, Edmonton, AB T6G 2S2, Canada

**Keywords:** pupil responses, biosignal, task load, task familiarity, gaze position, visual tracking

## Abstract

Pupil size is a significant biosignal for human behavior monitoring and can reveal much underlying information. This study explored the effects of task load, task familiarity, and gaze position on pupil response during learning a visual tracking task. We hypothesized that pupil size would increase with task load, up to a certain level before decreasing, decrease with task familiarity, and increase more when focusing on areas preceding the target than other areas. Fifteen participants were recruited for an arrow tracking learning task with incremental task load. Pupil size data were collected using a Tobii Pro Nano eye tracker. A 2 × 3 × 5 three-way factorial repeated measures ANOVA was conducted using R (version 4.2.1) to evaluate the main and interactive effects of key variables on adjusted pupil size. The association between individuals’ cognitive load, assessed by NASA-TLX, and pupil size was further analyzed using a linear mixed-effect model. We found that task repetition resulted in a reduction in pupil size; however, this effect was found to diminish as the task load increased. The main effect of task load approached statistical significance, but different trends were observed in trial 1 and trial 2. No significant difference in pupil size was detected among the three gaze positions. The relationship between pupil size and cognitive load overall followed an inverted U curve. Our study showed how pupil size changes as a function of task load, task familiarity, and gaze scanning. This finding provides sensory evidence that could improve educational outcomes.

## 1. Introduction

Wearable sensor technologies have advanced significantly to detect behavioral and physiological status in different task environments [1]. Pupil size/diameter monitoring is a reliable and valid evaluation approach that has been widely used in ergonomic and educational research, such as human–computer interactions [2]. Pupil size relates to underlying human behavioral information such as attention, arousal, and emotion [3]. Understanding changing pupil patterns in pupils may help promptly reveal information about the cognitive status [4]. Existing evidence has shown that pupil size generally increases with individuals’ cognitive load [5,6,7]. Nonetheless, previous research has primarily examined their relationship through a simple linear lens. It has been shown that such a simple linear relation may not sufficiently encapsulate the intricate pattern involved in pupil variation [8,9].

The Yerkes–Dodson Law posits that performance initially improves as stress or arousal levels increase until an optimal level of arousal is reached, beyond which further increases may impair performance [10,11]. Performance tends to decline when cognitive limits are exceeded. Nonetheless, assessing cognitive load according to each person’s performance is inherently retrospective. It is necessary to have a timely and objective assessment available for real-time monitoring of cognitive load [12]. Monitoring pupil size is an effective method for achieving this objective [13]. However, it is still unknown how pupil size quantitatively changes in response to task load during the transition from cognitive underload to overload. The extent to which a similar inverted-U correlation pattern can be observed between pupil size and cognitive load remains uncertain. 

Additionally, pupil size can be affected by complex factors. When doing a task, factors such as task familiarity [14] and gaze position [15] can also impact pupil size during learning. As participants scan different areas of the task interface, the features of the objects being viewed may lead to changes in pupil size [16]. Anticipatory eye movements may also lead to pupil size changes [16,17]. Questions remain as to how these factors may affect pupil size. Therefore, further research should be conducted to elucidate how task familiarity and gaze position can affect pupil size [6]. 

Our research aimed to investigate the effect of task load, task familiarity, and gaze position on pupil size changes during learning. We also investigated their potential interaction. We employed an arrow tracking task to capture the dynamic patterns of pupil size. The arrow tracking task was selected for its ability to systematically manipulate cognitive load while maintaining a controlled environment. We tracked pupil changes across a range of task loads, from low to very high. Task load was manipulated by progressively increasing path lengths and direction changes of arrow movements from block 1 to block 5. Each task was repeated twice to account for task repetition, which is indicative of task familiarity. Additionally, we delineated areas of interest (AOI) frame by frame to differentiate gaze positions, including eye before arrow (EBA), eye on arrow (EOA), and eye on other areas (EOO). Each position represented a distinct focus on the task interface.

Our study proposed three hypotheses. First, changes in task load will influence the participants’ pupil size, with an increase in pupil size corresponding to escalating task load until a threshold is reached, after which pupil size will decrease. Second, task repetition will lead to a decrease in pupil size. Third, gaze position will impact pupil size, with larger pupil sizes being observed when focusing on the area preceding the target compared to other areas. The null hypothesis posits that no significant differences in pupil size will be present across various factorial conditions. Our objective is to offer insights into the objective assessment of cognitive processes during learning. We aim to characterize the threshold at which cognitive overload transpires using pupil size, analyzed via a polynomial lens. These findings may provide pupil-based sensory evidence that has implications for educational practices, cognitive assessment, and interface design [18,19]. By identifying the cognitive threshold through pupil size changes, educators can tailor instructional materials to maintain optimal cognitive load, enhancing students’ learning experiences and improving educational outcomes [19].

## 2. Method

This repeated measures 2 × 3 × 5 (task repetition × gaze location × task difficulty) factorial [20] study was conducted at the Surgical Simulation Research Lab of the University of Alberta. This study was not preregistered. The Institutional Review Board of the University of Alberta approved the study, and participants were recruited from the campus community in 2022. Inclusion criteria were healthy adults with normal or corrected-to-normal vision. All 15 participants who provided written informed consent completed all possible within-subject conditions. The participants have an average ± standard deviation age of 28 years ± 7 years, consisting of five men and 10 women.

### 2.1. Experimental Setting

**Figure 1:**
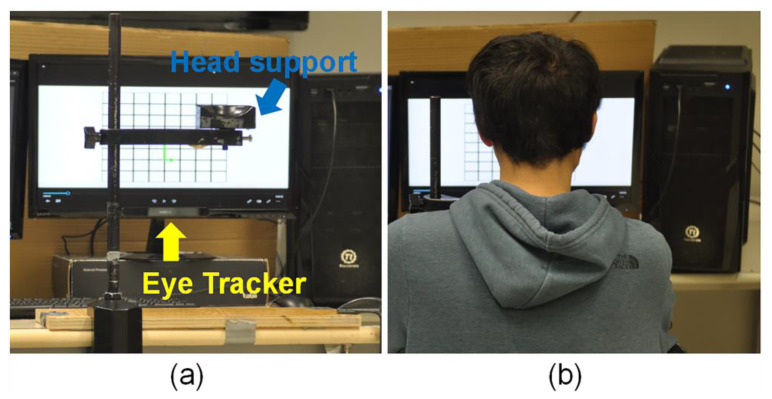
Experimental setting. Note: (**a**) the Tobii Pro Nano eye tracker is denoted by yellow text; the head support is denoted by blue text. (**b**) Participants were seated in front of the computer, positioning their chins on the designated support.

The experimental setting is demonstrated in Figure 1. The visual display was presented to participants via a standard personal computer (Intel, Santa Clara, CA, USA) connected to a monitor (ASUS Computer Inc., Taipei, Taiwan) measuring 476 mm by 370 mm. To minimize head motion and ensure the consistency of each repeated trial, participants’ head positions were stabilized at 65 cm from the display monitor with the aid of a support apparatus for the jaw (Figure 1). To eliminate any interference, factors such as room light and screen color that could influence pupil size were kept completely consistent for all participants in each test condition. Every participant was permitted to adjust the support structure to achieve a comfortable and optimal position. Pupil size data during the task were gathered using Tobii Pro Nano, a remote, screen-based eye-tracking system (Tobii Technology Inc., Stockholm, Sweden).

### 2.2. Task Design

A series of learning tasks with incremental task load was created. Participants were given an orientation before the actual experiment to ensure they understood the task requirements. In the experiment, they were asked to watch videos showing arrow movement in an 8-by-8 grid and memorize the movement patterns displayed. These patterns escalated in complexity, featuring three, five, seven, nine, and eleven turning points, respectively. Each video served as the stimulus for a corresponding task block, labeled block 1 through block 5. As the blocks progressed from 1 to 5, the task complexity increased incrementally, mirroring the rising complexity in the video stimuli. The length of each movement pattern in the videos, measured in cell lengths, was four times the number of turning points. The arrows in the videos consistently moved at a speed of one cell length per second. Therefore, the total time duration of each video, measured in seconds was calculated by multiplying the number of turning points by four cell lengths and then by dividing it by the movement speed of one cell length per second. To account for familiarity effects, each video was played twice (repetition), constituting trial 1 and trial 2 in this study. 

**Figure 2:**
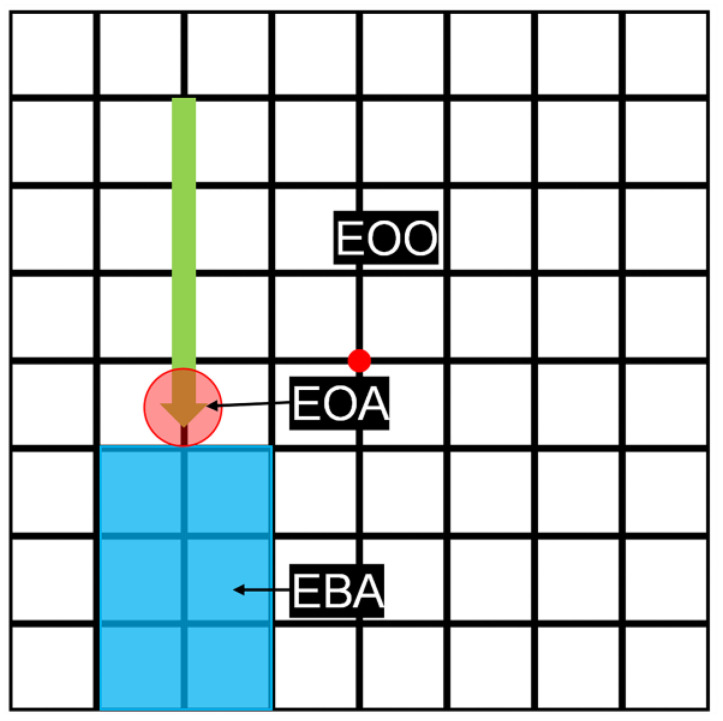
Illustration of Areas of Interest. Note: red regions represent the Eye on Arrow (EOA), blue regions represent the Eye Before Arrow (EBA), and the remaining areas indicate the Eye on Other Areas (EOO).

The AOIs manually defined by the experimenter are presented in Figure 2. The EOA was a circular region with a 100-pixel diameter, centered on the arrow tip. The EBA was a rectangular region extending two grid widths before the arrow, with its edges continuously touching the arrow tip and adjusting to the arrow movement. The EOO was the remaining area of the task interface, excluding the EOA and EBA. Pupil size measurements were recorded while the participant’s gaze was focused on each AOI. These measurements were calculated to obtain a mean value for each AOI. Consequently, for each participant, the experiment produced 2 × 3 × 5 or 30 measured pupil data points.

### 2.3. Task Procedures

Following orientation, participants sequentially viewed videos from block 1 to block 5. They were instructed to concentrate on the arrow direction and distance, and verbally recall the pattern immediately after each video. After completing each block, they had a 1 min break before taking the NASA-Task Load Index (NASA-TLX) assessment, which is a well-established rating scale for evaluating individuals’ cognitive load [21]. Adequate rest time between blocks was provided to minimize carryover effects (wait until pupil size stabilizes). This process was repeated until all five blocks were completed.

### 2.4. Cognitive Load and Performance Measures

The cognitive load was measured once per block, while the performance score was assessed twice in two trials per block. We used NASA-TLX to evaluate participants’ cognitive load across six domains: mental demands, physical demands, temporal demands, effort, frustration, and performance [21]. A custom-designed software was used to enable participants to rate each domain and respond to a series of paired-choice questions to determine each domain’s weight. The software automatically calculated an overall NASA-TLX score for each participant based on their domain ratings and weights, which was then used for further analysis.

During the verbal recall phase, participants’ responses were used to determine their performance scores. The experimenter evaluated each response by comparing it to the correct movement at every turning point, characterized as “distance-direction” (e.g., five units-up). Participants received one point for accurately recalling the movement in both distance and direction. To account for varying turning points across blocks, each participant’s points were divided by the total number of turning points within that block before being aggregated to generate a final performance score for data analysis. Performance scores ranged from 0 to 1, with 1 representing optimal performance.

### 2.5. Pupil Data Collection and Processing

**Figure 3:**
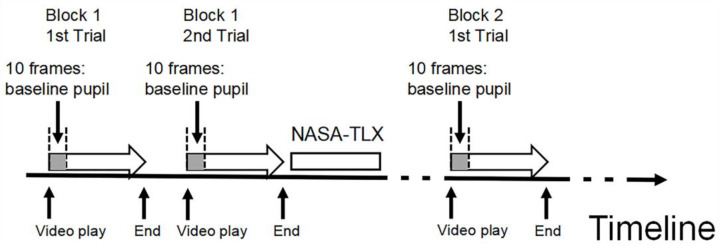
Illustration of baseline pupil size and measured pupil size. Note: for every task condition, the pupil size obtained from the initial ten sampling frames was averaged to determine the baseline pupil size. All remaining frames within that specific task condition were averaged to determine the measured pupil size. The sampling rate of pupil size was 60 Hz.

Participants completed the arrow tracking task while the eye tracker recorded their pupil data in real time at a 60 Hz sampling rate. Tobii Pro Lab processed and exported pupil data frame by frame. As shown in Figure 3, the baseline pupil size for each task condition was calculated by averaging the pupil size associated with the initial 10 frames before the arrow video started to play. The measured pupil size was obtained by averaging all remaining frames when each arrow video was playing. The adjusted pupil size, acquired by subtracting the baseline value from the measured pupil size, was recorded for each participant under every task condition for subsequent statistical analysis.

### 2.6. Statistical Analysis

Statistical analysis was performed using R (version 4.2.1) and R Studio. A three-way factorial repeated measures analysis of variance (ANOVA) assessed the main and interactive effects of task repetition, task load, and gaze position. Due to the difference in measurement frequency for the NASA-TLX score and performance score, two different statistical models were employed, that being one-way and two-way within-subjects ANOVAs, respectively. Test assumptions were carefully checked and satisfied. Data normality was assessed using the Shapiro–Wilk test, sphericity using the Mauchly’s test, and the Greenhouse–Geisser method accounted for sphericity violations [22]. Power analysis was conducted in R for each factor, utilizing partial eta squared as the effect size measure. Simple main effect analysis was executed for factors exhibiting significant interaction. We used the Bonferroni correction [23] to control inflated alpha levels when comparing all within-subject conditions in post hoc analyses.

To further investigate the association between cognitive load and pupil size, we employed a linear mixed-effect model using the lme4 package [24] in R. The model employed pupil size as the dependent variable, NASA-TLX scores as fixed effects independent variables, and participants as random effects. Additionally, polynomial contrasts were performed to further scrutinize the trend. We compared linear, quadratic, cubic, and quartic models based on task load levels to determine the model offering the most accurate pupil size estimates. The Residual Sum of Squares (RSS) served as a criterion for model selection [25], with the lowest RSS value indicating the best fit due to minimized unexplained variance in the data. A *p*-value < 0.05 was considered statistically significant.

## 3. Results

### 3.1. Sample Information & Data Examination

Fifteen participants yielded a total of 450 adjusted pupil data points, where each participant performed two trials on each of the five video tracking tasks. For each task, pupil data were calculated from three different AOIs (EBA, EOA, and EOO). The data met the necessary test assumptions, including equal subgroup sizes and normality. Two extreme outliers were identified and adjusted using the winsorization method. Table 1 presents the marginal means (standard error) of the adjusted pupil size under various conditions. Mauchly’s test indicated that the assumption of sphericity had not been met. To adjust for the lack of sphericity, we exported the adjusted *p*-values using the Greenhouse–Geisser correction.

### 3.2. Power Analysis

The effect sizes, measured as partial eta squared, were 0.058 for task load, 0.143 for repetition, and 0.0002 for gaze position. The power analysis revealed a power of 0.81 for task load and nearly 1.00 for task repetition at a significance level (α) of 0.05. However, the effect size for gaze position was quite small, suggesting a negligible impact on pupil size. The study found that the dataset from 15 participants, which provided a total of 450 data points, was adequate for detecting significant effects of task load and repetition within the framework of a three-way repeated measures ANOVA. The analysis included five levels of task load, two levels of task repetition, and three levels of gaze position.

### 3.3. Cognitive Load

A one-way within-subjects ANOVA was conducted to examine the effect of task load on cognitive load or NASA-TLX scores, revealing a significant main effect (F = 64.82, *p* < 0.001). The NASA-TLX scores significantly differed among block 1 (17.82 ± 13.04), block 2 (34.16 ± 17.98), block 3 (52.87 ± 20.04), block 4 (59.91 ± 16.44), and block 5 (72.91 ± 15.54). Post hoc analysis indicated statistically significant differences (*p* < 0.05) between almost all pairs of blocks, except for blocks 3 and 4 (F = −1.86, *p* = 0.08). There was an overall increase in cognitive load from the first to the fifth task block.

### 3.4. Performance

**Figure 4:**
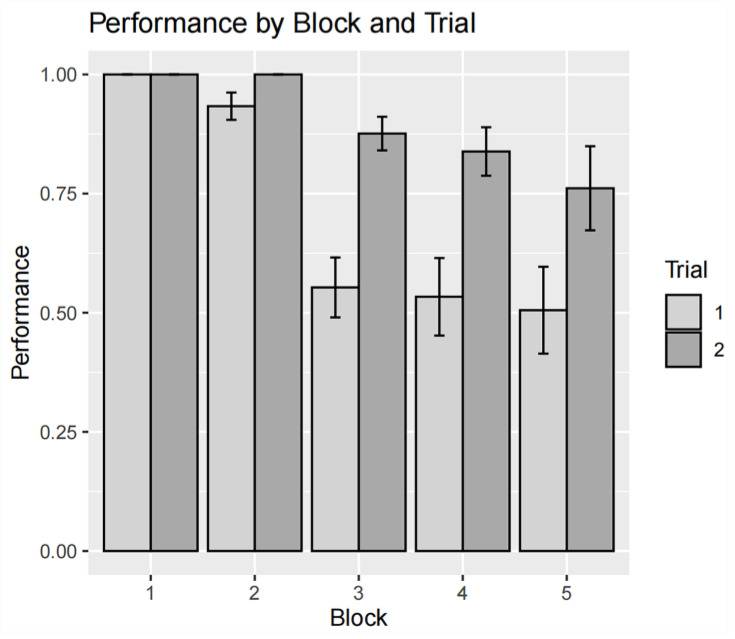
Interaction plot displaying the influence of block and trial on the participants’ performance scores. Note: error bars represent standard errors of the performance score for each group.

A two-way within-subjects ANOVA was conducted on performance scores, revealing significant main effects for both block (F = 18.93, *p* < 0.001) and trial (F = 62.35, *p* < 0.001), as well as for their interaction (F = 14.74, *p* < 0.001). The effect of task repetition was significant only in block 3 (F = 47.40, *p* < 0.001), block 4 (F = 23.20, *p* < 0.001), and block 5 (F = 33.90, *p* < 0.001). Figure 4 illustrates the interaction of performance between two repeated trials and five task blocks.

### 3.5. Pupil Size

**Figure 5:**
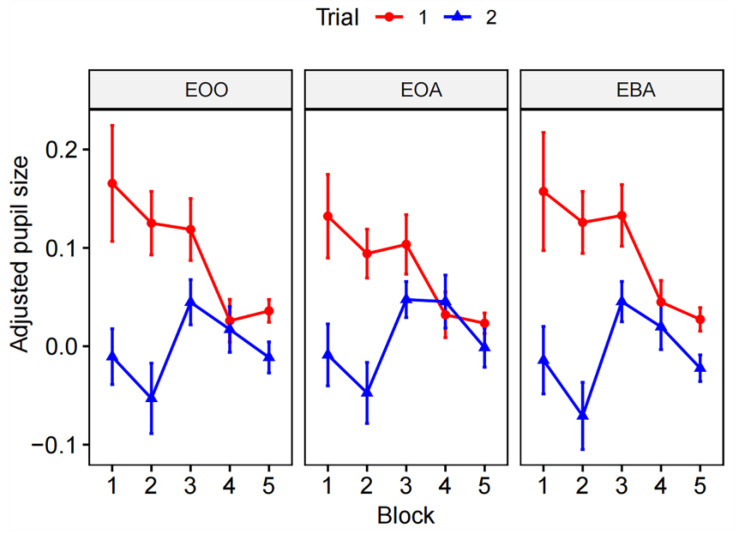
Interaction effects in a three-way repeated measures ANOVA. Note: this plot illustrates the interaction effects of task load (x-axis, represented by varying blocks), repetition (unique colored lines), and gaze position (three faceted subgraphs representing different AOIs) on the dependent variable, adjusted pupil size. Faceted subgraph EOO, Eye on Other Areas, EOA, Eye on Arrow, EBA, Eye Before Arrow. Significant interactions among variables are indicated by non-parallel lines. Error bars represent standard errors of the adjusted pupil size for each group.

A three-way factorial repeated measures ANOVA was conducted to compare the main effect of task load, task repetition, gaze position, and their interaction effects on pupil size. Table 2 displays the results. We found a statistically significant main effect of task repetition on adjusted pupil size (F (1, 14) = 19.75, *p* < 0.001, partial η^2^ = 0.143). A borderline significant effect of task load was also observed (F (4, 56) = 2.36, *p* = 0.064, partial η^2^ = 0.058), suggesting a small overall difference in pupil size across different task difficulties. There were no statistically significant differences in pupil size between gaze positions. Significant interaction effects were only observed between task load and repetition (F (2.38, 33.28) = 5.42, *p* = 0.006, partial η^2^ = 0.081) and between task repetition and gaze position (F (2.00, 28.00) = 4.27, *p* = 0.006, partial η^2^ = 0.005). The interactions are graphically displayed in Figure 5. It suggests that mean pupil sizes generally decreased as the task load increased, with more pronounced differences between trial 1 and trial 2 at lower task loads.

Significant interactions were identified between task repetition and task load, as well as between task repetition and gaze position. To further elucidate the nature of these interaction effects, a simple main effect analysis was conducted. In terms of the interaction between task repetition and task load, significant repetition effects were observed for block 1 (F(1, 14) = 7.79, *p* = 0.014, partial η^2^ = 0.194), block 2 (F(1, 14) = 22.8, *p* < 0.001, partial η^2^ = 0.344), block 3 (F(1, 14) = 8.74, *p* = 0.01, partial η^2^ = 0.119), and block 5 (F(1, 14) = 11.5, *p* = 0.004, partial η^2^ = 0.127), but not for block 4 (F(1, 14) = 0.072, *p* = 0.792, partial η^2^ = 0.002). Regarding various gaze positions, significant repetition effects were detected for EBA (F (1, 14) = 19.8, *p* < 0.001, partial η^2^ = 0.172), EOA (F (1, 14) = 15.0, *p* = 0.002, partial η^2^ = 0.106), and EOO (F (1, 14) = 17.8, *p* < 0.001, partial η^2^ = 0.172).

A post-hoc analysis was conducted to discern significant differences among specific groups, with the Bonferroni correction applied to mitigate inflated Type I error (α). Concerning the effect of task repetition, a significant difference was observed between trial 1 (0.09 ± 0.13) and trial 2 (0 ± 0.1) in terms of pupil size (*p* < 0.001). In the context of task load, significant disparities emerged between block 1 (0.07 ± 0.19) and block 5 (0.01 ± 0.06) (*p* = 0.02), block 3 (0.08 ± 0.11) and block 2 (0.03 ± 0.15) (*p* < 0.001), block 3 and block 4 (0.03 ± 0.09) (*p* = 0.01), and block 3 and block 5 (*p* < 0.001). These results suggest that pupil size may decrease in response to task repetition, while increased task load may yield inconsistent alterations in pupil size. 

### 3.6. Linear Mixed-Effect Regression

We separately analyzed the data of trial 1 and trial 2 since there were significantly different trends. Our study found that the correlation between NASA-TLX and pupil size was not simply linear. To determine the most suitable regression model for each trial, we compared linear, quadratic, cubic, and quartic models. The quartic regression was the best fit for trial 1 and the entire dataset, while a quadratic model was more appropriate for trial 2. For trial 1, the optimal regression model was: pupil size~poly (NASA-TLX, 4) + (1 + poly (NASA-TLX, 4)|participant), with R-squared values of 0.072 for fixed-effects factors and 0.290 for random-effects factors. The optimal regression model for trial 2 was: pupil size~poly (NASA-TLX, 2) + (1 + NASA-TLX|participant), with R-squared values of 0.098 for fixed-effects factors and 0.604 for random-effects factors. While the correlation pattern between NASA-TLX and pupil size differed between trial 1 and trial 2, NASA-TLX can account for a visible and reasonable portion of the variation in both trials. Individual differences (i.e., the random effect of participant) played a substantial role in explaining the variance of pupil size in both trials.

**Figure 6:**
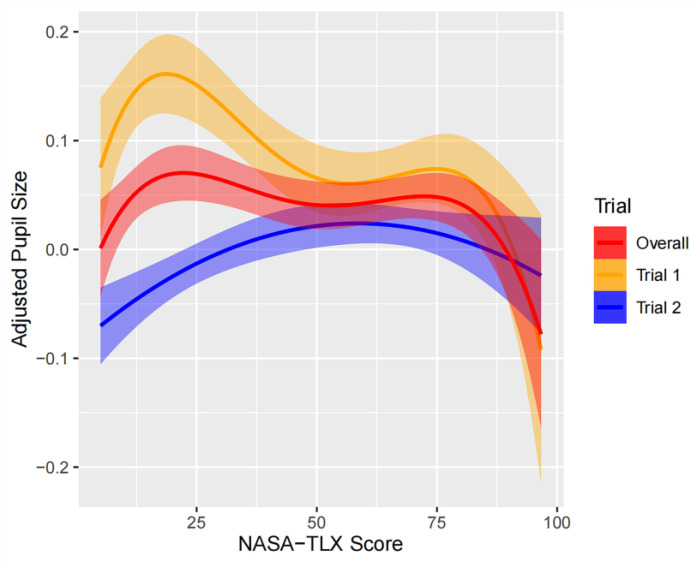
Linear mixed-effects regression plot. Note: this plot displays the relationship between adjusted pupil size and NASA-TLX scores using a linear mixed-effects regression model, accounting for participants as a random effect. Different colored lines represent individual trials or the overall trend. The shaded area represents the 95% confidence interval for the regression line.

Figure 6 depicts the quadratic model, and, notably, in trial 2, an inverted-U curve pattern is evident in pupil size changes as the NASA-TLX score rises. These findings indicate that a more complex model is necessary to accurately capture the relationship between cognitive load and pupil size.

## 4. Discussion

Our study aimed to explore pupil size changes as a function of task load, familiarity, and gaze searching area during a learning task of visual tracking. The findings provided insights into the pupil response during learning and have implications for task load management in educational settings.

To establish construct validity, we first analyzed the NASA-TLX and performance scores under various task conditions. Our results align with the intended effects of the experimental design. There was a steady and significant increase in participants’ cognitive load from block 1 to 5, as assessed by the NASA-TLX. In Figure 4, it can be seen that the performance decreases with each successive block. Beginning with block 3, participants were unable to fully manage the tasks. Performance score gaps between trials were significantly larger in task blocks 3, 4, and 5 compared to blocks 1 and 2. The pattern observed in participants’ performance suggests that the threshold separating the transition from cognitive underload to cognitive overload lies between task blocks 2 and 3.

Task familiarity effect was tested by repeating the task using the same movement pattern. In line with our second hypothesis, we observed a significant effect of task repetition on pupil size, with notably smaller pupil sizes in the second trial. This phenomenon can be explained by the fact that individuals gain familiarity or experience with a task through repetition. The participants’ cognitive load and mental effort were reduced, which led to reduced pupil size [14,26]. Furthermore, this effect was only present in the initial blocks 1–3 and became less pronounced in the more challenging blocks 4–5. As the tasks became more complex, participants could not achieve the same level of automaticity with a single repetition as with simple tasks, resulting in a negligible reduction in pupil size.

Regarding our third hypothesis, the eye pupil did not show a significant difference among the three gaze positions. This phenomenon may be attributed to several factors. First, it could be due to the nature of the visual tracking task. Early eye disengagement may not be more important for the visual tracking than other types of tasks requiring hand manipulation. Therefore, the gaze on the area before the arrow failed to elicit discernible pupil responses relative to the gaze on the arrow and other area. Second, rapid eye movements among these gaze areas may not provide sufficient time for the pupil response to elicit significantly different pupil sizes among the three AOIs. In the future, we will examine pupil response when eyes are scanning over different target areas in tasks that require hand manipulation. 

Our first hypothesis proposed an inverted U-shaped correlation between task load and pupil dilation. This hypothesis was supported in trial 2. In contrast, in trial 1, block 1 recorded a significantly larger pupil size, a phenomenon possibly due to the participants’ first encounter with the tasks. This first encounter may have increased cognitive arousal. Specifically, the unfamiliarity of the task may have triggered an elevated level of cognitive load that disproportionately affected pupil size, resulting in a deviation from the expected U-shaped pattern. However, in trial 2, familiarity with the tasks through repetition reduced the effect of unfamiliarity, allowing for a clearer demonstration of the predicted inverted U-shaped response. This result was consistent with our hypothesis, but also highlighted the complex dynamics between human cognition and pupil responses. It suggests that further investigation of initial pupil adaptations in response to novel tasks may be required.

In addition, our analysis used a linear mixed-effects model to dissect the relationship between cognitive load and pupil size, accounting for individual variances [27]. This nuanced approach revealed a more pronounced inverted U-shaped trend in the second trial, consistent with the claims of the Yerkes–Dodson law [10,11]. These findings highlight the feasibility of monitoring pupil size to identify individual cognitive limits, thereby facilitating the adjustment of task load to improve individual performance [12,28].

This study has some limitations. The first limitation is associated with the eye-tracking technology itself. Although the eye-tracker device that we used has been widely utilized in research studies, there is still some level of inaccuracy associated with its use. For instance, the accuracy and precision of Tobii Pro Nano are 0.3° and 0.1°, respectively, which can impact the quantification of pupil location. In addition, despite controlling the experimental setting, individual differences, such as comfort with the device, can introduce variability during data collection. The second is that the visual tracking task findings might not fully represent behavior patterns across the diverse tasks in learning goal-directed movements, which can limit the generalizability of the finding.

Further research should aim to enhance the precision and accuracy of eye-tracking technology and explore the relationships among pupil size, cognitive load, and performance in various learning contexts that include hand motions and decision making as part of the task. Examples of such exercises include tasks involving decision making [6], calculation [29], or eye–hand coordination [28,30]. This research holds significant potential for application in education and various other fields, particularly those demanding high levels of skill and cognitive capacity, such as surgery and aviation [2,31]. Pupil size serves as an informative biosignal, offering real-time, non-invasive insights into the cognitive states of trainees. Knowledge gained from our study can contribute to the creation of adaptive and supportive training and working systems based on pupil signals.

## 5. Conclusions

The study investigated how pupil size was influenced by task load, task familiarity, and gaze position during visual tracking tasks. Pupil diameter decreased with task familiarity. We also identified a characteristic inverted U-shaped pattern in pupil size as subjects moved from cognitive underload to overload. Pupil size was not influenced by gaze position. These results suggest that pupil size, as a biosignal, may serve as a reliable marker of cognitive assessment, providing opportunities to improve the learning experience.

## Figures and Tables

**Table 1:** Mean (standard errors) of pupil size across different test conditions.

Conditions	EOO + Trial 1	EOO + Trial 2	EOA + Trial 1	EOA + Trial 2	EBA + Trial 1	EBA + Trial 2
Block 1	0.17 (0.06)	−0.01 (0.03)	0.13 (0.04)	−0.01 (0.03)	0.16 (0.06)	−0.01 (0.03)
Block 2	0.13 (0.03)	−0.05 (0.04)	0.09 (0.02)	−0.05 (0.03)	0.13 (0.03)	−0.07 (0.04)
Block 3	0.12 (0.03)	0.04 (0.02)	0.10 (0.03)	0.05 (0.03)	0.13 (0.03)	−0.03 (0.03)
Block 4	0.03 (0.02)	0.02 (0.02)	0.03 (0.02)	0.05 (0.03)	0.04 (0.02)	−0.02 (0.03)
Block 5	0.04 (0.01)	−0.01 (0.02)	0.02 (0.01)	0 (0.02)	0.03 (0.01)	−0.04 (0.02)

Note. EBA, eye before arrow; EOA, eye on arrow, and EOO, eye on other areas.

**Table 2:** Three-way repeated measures ANOVA for task load, repetition, and gaze position.

Factors	DFn	DFd	F	*p*	*p* < 0.05	η^2^
Load	4.00	56.00	2.36	0.064		0.058
Repetition	1.00	14.00	19.75	<0.001	*	0.143
Gaze position	2.00	28.00	0.16	0.850		<0.001
Load × Repetition	2.38	33.28	5.42	<0.001	*	0.081
Load × Gaze position	3.32	46.45	0.82	0.500		0.003
Repetition × Gaze position	2.00	28.00	4.27	0.024	*	0.005
Load × Repetition × Gaze position	3.43	47.95	0.19	0.921		<0.001

Note: DFn, degrees of freedom numerator; DFd, degrees of freedom denominator. *, Statistical significance.

## Data Availability

The datasets analyzed in the current study are available from the corresponding author upon reasonable request.
